# Computer Adaptive vs. Non-adaptive Medical Progress Testing: Feasibility, Test Performance, and Student Experiences

**DOI:** 10.5334/pme.1345

**Published:** 2024-07-26

**Authors:** Elise V. Van Wijk, Jeroen Donkers, Peter C. J. De Laat, Ariadne A. Meiboom, Bram Jacobs, Jan Hindrik Ravesloot, René A. Tio, Cees P. M. Van Der Vleuten, Alexandra M. J. Langers, Andre J. A. Bremers

**Affiliations:** 1 Center for Innovation in Medical Education, Leiden University Medical Center, the Netherlands; 2 School of Health Professions Education, Faculty of Health, Medicine and Life Sciences, Maastricht University, the Netherlands; 3 Department of Pediatrics, Erasmus Medical Center, Rotterdam, the Netherlands; 4 Department of General Practice and Elderly Care Medicine, Amsterdam University Medical Center, Amsterdam, the Netherlands; 5 Department of Neurology, University of Groningen, University Medical Center Groningen, Groningen, the Netherlands; 6 Department of Physiology, Amsterdam University Medical Center, Amsterdam, the Netherlands; 7 Department of Cardiology, Catharina Hospital Eindhoven, Eindhoven, the Netherlands; 8 Department of Educational Development and Research, Faculty of Health, Medicine and Life Sciences, Maastricht University, Maastricht, the Netherlands; 9 Department of Gastroenterology and Hepatology, Leiden University Medical Center, Leiden, the Netherlands; 10 Department of Surgery, Radboud University Medical Center, Nijmegen, the Netherlands

## Abstract

**Background:**

Computerized adaptive testing tailors test items to students’ abilities by adapting difficulty level. This more efficient, and reliable assessment form may provide advantages over a conventional medical progress test (PT). Prior to our study, a direct comparison of students’ performance on a computer adaptive progress test (CA-PT) and a conventional PT, which is crucial for nationwide implementation of the CA-PT, was missing. Therefore, we assessed the correlation between CA-PT and conventional PT test performance and explored the feasibility and student experiences of CA-PT in a large medical cohort.

**Methods:**

In this cross-over study medical students (n = 1432) of three Dutch medical schools participated in both a conventional PT and CA-PT. They were stratified to start with either a conventional PT or CA-PT to determine test performance. Student motivation, engagement and experiences were assessed by questionnaires in students from seven Dutch medical schools. Parallel-forms reliability was assessed using the Pearson correlation coefficient.

**Results:**

A strong correlation was found (0.834) between conventional PT and CA-PT test performance. The CA-PT was administered without system performance issues and was completed in a median time of 83 minutes (67–102 minutes). Questionnaire response rate was 31.7% (526/1658). Despite a higher experienced difficulty, most students reported persistence, adequate task management and good focus during the CA-PT.

**Conclusions:**

CA-PT provides a reliable estimation of students’ ability level in less time than a conventional non-adaptive PT and is feasible in students throughout the entire medical curriculum. Despite the strong correlation between PT scores, students found the CA-PT more challenging.

## Introduction

In the mid-1970s, Maastricht medical school introduced the progress test (PT) to align the assessment system with the rationale of the innovative instructional method of problem-based learning. This initiative aimed to mitigate the test-directed learning stimulated by end-of-unit assessments [1]. By introducing this comprehensive test, which aims to assess the end objectives of the medical curriculum, specific test preparation was discouraged. Its longitudinal design together with the feedback enhances the educational impact, by fostering long-term learning of functional knowledge [2,3,4,5,6,7]. To ensure a valid and reliable test content, the Dutch PT uses a blueprint containing a prescribed distribution of items across medical classifications and disciplines [8]. When the study was conducted the PT was implemented in several countries as a paper- or computer-based test, consisting primarily of multiple-choice questions (MCQs) [3]. Today, students of all Dutch medical schools participate in a national PT [9]. As in this fixed linear test format the knowledge level of individual students is not considered, the test contains items at a distance from the students’ ability, which is likely to lower the test’s reliability; an important criterium for good assessment [7,10,11,12]. Furthermore, with the increasing number of participating medical schools during the past years, the simultaneous administration of progress tests to all students nationwide has become a logistical and costly challenge, limiting the feasibility of the test [7,12].

Computerized adaptive testing (CAT) is a form of digital assessment that delivers a more tailored test to individual students by adapting the questions to the examinee’s ability level using a pre-determined algorithm [13]. Usually, CAT adapts the difficulty level of the questions to the performance of the student during the exam. However, there are also other forms of computer adaptive testing available, each with their own assumptions, merits, and limitations. Some examples are multidimensional CAT [14], content-based CAT [15], testlet-based CAT [16], and tree-based CAT [17]. Our focus is on a CAT that is based on plain Item Response Theory (IRT), which is a cornerstone of modern test theory. Unlike the Classical Test Theory (CTT), which is the underlying theory of the conventional Dutch PT’s fixed linear test format, it does not assume that each question (or item) is equally difficult. Instead, it uses mathematical models to estimate the underlying ability level (‘theta’) of the test-taker based on their responses to different items. Each item is characterized by parameters that reflect its difficulty and discrimination, which allows for the creation of tests that are tailored to the test-takers ability level [18]. CTT on the other hand assumes that the observed total test score equals the actual ability level of the test-taker (‘true test score’) with an identical measurement error for all scores. These assumptions can lead to less precise estimates of a test-taker’s ability [19]. As such, the CAT provides a more efficient test by reducing test length on average by 50% while preserving or even improving the reliability of the test [10,11,20,21]. Moreover, the Online Adaptive International Progress Test (OAIPT) project showed that the adaptive test was well-accepted by students and might improve motivation and engagement, which was also demonstrated earlier in elementary and high school students [10,22,23]. Effective development and feasibility of implementing a computer adaptive PT (CA-PT) in medical education across several European countries has been demonstrated before [11]. Simultaneous test administration, to prevent fraud by sharing exam information, is no longer required with the use of an online tailored test, reducing logistical issues, and improving feasibility.

Considering the benefits of CAT, it has been considered as a promising alternative for the CTT-based fixed linear test format of the conventional Dutch PT. While several studies have demonstrated strong correlations between fixed-length short forms and CAT in patient-outcome measurements [24,25,26], there is a lack of research comparing test performance on a linear-fixed PT with a CA-PT; a comprehensive, longitudinal test that adapts to the ability level of the student, administered to students at various curricular ages. A direct comparison between a CA-PT and conventional PT, in the same cohort of students and in an authentic setting, has yet to be conducted. This comparison is a necessary step towards the ambitious goal of implementing the CA-PT at a national level across all medical schools. Therefore, we aimed to 1) evaluate the correlation between test performance on a CA-PT and a conventional PT, and 2) assess the feasibility and student experiences of a CA-PT in a large cohort of Dutch medical students who were offered both a conventional PT and CA-PT.

## Methods

### Setting

The Dutch interuniversity medical PT is a longitudinal comprehensive test that covers the whole medical curriculum. In the Netherlands, the medical curriculum consists of a preclinical Bachelor and clinical Master phase, both with an average duration of three years each. The preclinical phase is made up of a variety of theoretical courses. Each of these courses is assessed by a summative assessment to evaluate a student’s knowledge. The clinical phase is primarily composed of clinical rotations, which are separately or collectively evaluated by a summative pass/fail decision based on feedback from supervisors. The learning outcomes of the medical curriculum are described in a Framework for Undergraduate Medical Students, and are identical for all medical schools [27]. At the time of the study, seven of the eight Dutch medical schools participated in the PT. Throughout the six-year medical program, the PT is administered four times each academic year (September, December, February, and May), resulting in a total of 24 test moments for an individual student. The longitudinal design provides insights into a student’s functional knowledge development over time in relation to peer medical students across the Netherlands. The conventional non-adaptive PT consists of 200 MCQs and is identical for all participating students. The questions are selected from an item bank based on a blueprint with a predetermined distribution covering all relevant medical disciplines and categories (**Supplemental Table 1**). The MCQs include a “I don’t know” option symbolized by a question mark. Selection of this option results in a neutral score of zero points. An incorrect answer, on the other hand, incurs a penalty that results in a negative score. This so called formula scoring method encourages students to recognize their knowledge gaps and discourages random guessing [28]. The severity of the penalty of an incorrect answer is determined by the number of answer options. For instance, an incorrect answer in a MCQ with three options leads to a deduction of 1/3 points. This ensures that the penalty is proportional to the probability of guessing the correct answer. The final score is computed as the sum of the scores per MCQ and is expressed as a percentage of the maximum attainable score, and is translated into “*Good”, “Pass”*, or “*Fail”*, based on the mean and standard deviation of the complete student cohort in the same test moment as a relative standard. Progress in academic years goes along with increased passing scores of the PT. At the end of each academic year, the results of the four formative progress tests are combined into a summative decision (fail, pass, or good) [9].

### Development of the question bank

At the time of the study, the CA-PT item bank consisted of 3400 calibrated questions. These questions originate from 30 previous linear progress tests, spanning a period of 7.5 year. All questions were reviewed according to a rigorous peer-review process to determine if they were still correct and up-to-date before adding them to the item bank. Using the answer data from these historical 30 tests, we calibrated these questions following a Rasch model, a widely used IRT approach, to obtain their difficulties [29]. Question pairs assessing the same topic in a textual similar way, and conflicting questions were classified as enemy items, meaning that the system prevents usage of these questions in a single test. Before the questions had been used in a PT, they received a label for “*Category*” and “*Discipline*”, which places them in individual cells of the blueprint (**Supplemental Table 1**).

### Question selection in the CA-PT

The CA-PT consists of 135 MCQs without a question mark option; 120 calibrated questions, and 15 non-adaptive pretest questions. Every student receives questions according to the PT blueprint (**Supplemental Table 1**).The decision to use a fixed number of 120 questions was driven by our objective to reduce the overall length of the PT while still sufficiently covering the blueprint. We use a fixed-length CAT to provide a similar test experience for all students. The pretest questions are seed items (newly written or revised questions), randomly distributed throughout the CA-PT, are included for calibration, and do not contribute to the test result. After calibration, these new questions are added to the item bank for subsequent use. Prior to the adaptive phase of the CA-PT (i.e., 114 questions), six non-adaptive calibrated starter questions are administered to make a first estimation of the student’s ability level. The average difficulty level of these six questions together is zero, and the questions count for the test result. Due to the adaptive nature of the CA-PT, navigation is only unidirectional, whereas in the conventional PT students had the possibility to review previously answered questions during the test and change their answer if desired. The score of the CA-PT is the estimated ability level based on the answers on the 120 calibrated questions selected by the algorithm combined with the item difficulty of the questions [30].

### Study design and data collection

In this cross-over study students participated in both a conventional PT and CA-PT in May 2022, which was the last PT of the academic year 2021–2022. The conventional PT was mandatory for all students, and participation in the CA-PT was voluntary. To encourage students to perform at their best in both tests, the highest outcome was taken into account for their study progress. Students were stratified to start with either a conventional PT (PT*first*) or a CA-PT (CA-PT*first*) based on a fixed availability of the timeslots for each test moment. The conventional PT was administered as a paper-based test and the CA-PT as a digital test in TestVision®. Both PTs were administered in an exam hall with supervision. The conventional PT was administered to all students on the same day, during the same time slot. The allotted time to complete the PT was 240 minutes for the conventional PT, and 180 minutes for the CA-PT. The time interval between the conventional PT and the CA-PT for an individual student was seven days or less. The test results were communicated to students by email after two weeks for the conventional test, and after five weeks for the CA-PT.

On completion of the CA-PT, digital questionnaires were administered to gain insights into the student experiences (**Supplemental Material 2**). All students had previous experience with the paper-based PT. At the time of administration of the questionnaire, students were unaware of their test results. Items 1–11 of the questionnaire were derived from the Short Motivation and Engagement Scale (six items on positive, and five items on negative test-relevant motivation and engagement), adapted to our context and translated to Dutch [31]. Items 12–15 assessed the subjective experience of the CA-PT in comparison to the conventional PT, and were based on the questionnaire used in the study by Martin & Lazendic [22]. Five out of the seven items were found relevant to include in our questionnaire.

### Participants

Students from all participating medical schools were offered the opportunity to participate in a CA-PT of May 2022. In three of the participating medical schools (MS1, MS2, and MS3) the CA-PT could be offered to all students under full study conditions. Due to logistic issues and/or a lack of approval by the local board of examiners, students from the other four medical schools were not able to participate in the study, although some students had the opportunity to try-out the CA-PT without the result being taken into account. Students who participated in both a CA-PT and conventional PT in MS1, MS2, and MS3 were included for analyses regarding *test performance*. Regarding *feasibility* of CA-PT administration, and *student experiences*, we analyzed the data of all participants of the seven medical schools. The PT in May 2022 (the fourth PT of the academic year) entailed test moments 4 (year 1), 8 (year 2), and 12 (year 3) for the bachelor students and 13 to 24 for the master students, as master students enter the master phase at different timepoints throughout the year. For master students in Erasmus MC, this was only test moment 13 to 16, as the PT was introduced there in September 2021 for the master.

Information materials about the CA-PT were developed on a national level, and used by all medical schools. There were short animations about the CA-PT (see for example: https://www.youtube.com/watch?v=xjwHLhXhIho), written information, and frequently asked questions on the Dutch PT-website [32]. A national webinar for students was organized and recorded for later use. Furthermore, individual medical schools communicated identical information with their students via their local communication systems and/or organized (web)lectures.

### Data analysis

To assess possible differences in PT scores between PT*first*, and CA-PT*first* of the three participating medical schools we used z-scores, and an unpaired t-test. We also compared the z-scores of students who participated in our study with the z-scores of students who only participated in the conventional PT. The z-scores were calculated for the conventional PT, and CA-PT relative to all students in the same test moment group, providing a level of each student relative to their peers. Effect sizes were determined by the Cohen’s d coefficient. The Pearson correlation coefficient was utilized to evaluate the correlation between the total score on the conventional PT, and the theta (ability level) [33] on the CA-PT across both tests. The total score of the conventional PT was selected for this analysis, as this includes the question-mark option in the score. This question-mark option, and thereby the decision to answer a question or not, is an essential part of the conventional PT. Consequently, this approach provided the most reliable and authentic method for comparing the different PT formats. Characteristics of responders to the questionnaires are presented as mean (standard deviation), or median (interquartile range) depending on their distribution. Categorical variables are presented as number (proportion). All statistical analyses were performed using R version 4.1.0 (R Foundation for Statistical Computing, Vienna, Austria).

### Ethical approval

The approval to conduct this study was granted by the Ethical Review Board of the Netherlands Association for Medical Education (NVMO): NERB/2023.4.6. Participation in the CA-PT was voluntary, and all students received verbal and written information prior to the study. Upon initiation of the CA-PT, students provided informed consent.

## Results

**Figure 1:**
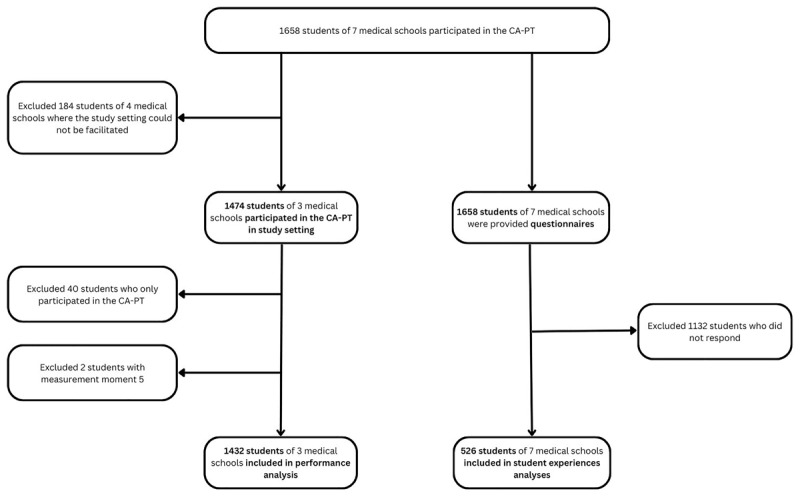
Flowchart of participants in questionnaire and test performance analyses.

In total 1432 students (647 bachelor, 785 master) from MS1, MS2, and MS3 were included in our analysis regarding student performance. In the other medical schools, a total of 226 students took part in the CA-PT, but their test results were not taken into account in the performance calculations as the study conditions were not met. Of the 1658 participating students in all medical schools, 526 students (response rate 31.7%) completed the questionnaire (Figure 1).

### Test performance

**Figure 2:**
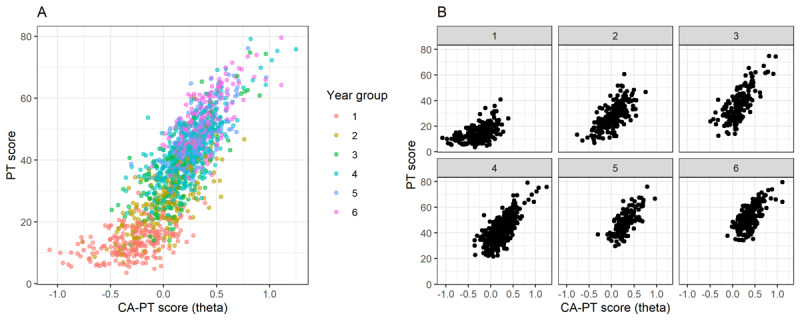
The relationship between the z-scores on the conventional PT (y-axis) and the theta on the CA-PT (x-axis) for **A)** year 1 to 6 and for **B)** each year separately.

Students in the PT*first*-group (*n* = 797; mean (M) = 0.406, SD = 1.06) performed slightly better on the conventional PT compared to students in the CA-Pt*first*-group (*n* = 635; M = 0.24, SD = 1.03; t(1373) = 3.08; p = 0.002; Cohen’s d = 0.16). No difference was found in performance on the CA-PT between both groups (t(1345) = –1.0324, p = 0.302). Within the three participating medical schools there was a small but significant difference between the conventional PT scores of students who participated in both a conventional PT and CA-PT, and students who participated only in a conventional PT in MS1 (M = 0.38, 0.19, SD = 1.09, 0.98; t(1444) = 3.49, p < 0.001; Cohen’s d = 0.18), and MS2 (M = 0.28, 0.16; SD = 0.99, 0.93; t(738) = 2.24; p = 0.025; Cohen’s d = 0.13), but not in MS3 (t(551) = 0.59, p:0.551). The parallel-forms reliability, i.e. the correlation between the total score of the conventional PT, and the theta of the CA-PT was 0.834. After adjustment for the differences in PT score between PT*first* and CA-PT*first* the correlation becomes slightly less: 0.832. The correlation was moderate within each year group: 0.506 (Y1; *n* = 253), 0.675 (Y2; *n* = 211), 0.754 (Y3; *n* = 183), 0.733 (Y4; *n* = 414), 0.708 (Y5; *n* = 164), and 0.673 (Y6; *n* = 207) (Figure 2).

### Feasibility of the CA-PT

Ninety percent of students finished the CA-PT within two hours (median: 83 minutes; IQR: 67–102 minutes). There were no performance issues with the digital assessment system. The algorithm was able to select questions of an appropriate level, defined as a difference of less than 0.1 between the estimated ability of the student, and pre-calibrated level of difficulty of the question, in more than 99% of the questions.

### Student motivation, engagement, and experiences

**Figure 3:**
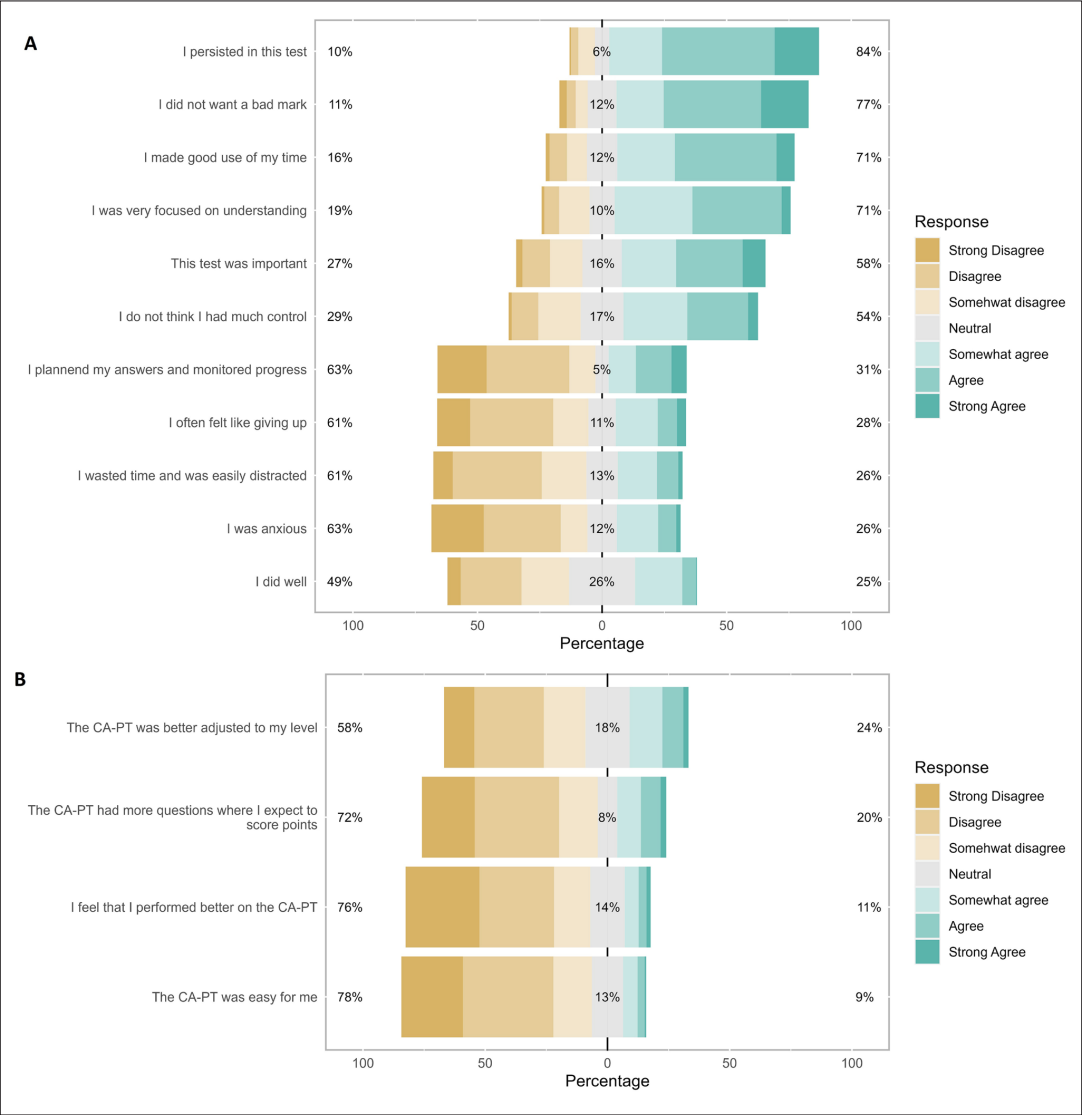
Distribution of answers to the questionnaire items **A)** 1 to 11 on motivation and engagement in the computer adaptive progress test; **B)** 12 to 15 comparing the conventional progress test with the computer adaptive progress test. I persisted in this test = ‘I persisted in this test even when it was challenging or difficult.’

Of the 526 responders to the questionnaire, 451 students were from MS1, 2, and 3. The responders had a mean age of 22.8 (3.0) years, and 74.1% were female. The median test moment was 15 (IQR: 8–19). Eighty-four percent of the students agreed that they persisted even when the CA-PT was challenging or difficult. Most students did not want to receive a bad grade for this exam (77%), made good use of their time during the CA-PT (71%), and were focused on understanding the questions (71%). The majority of students were not anxious (63%) or felt like giving up during the CA-PT (61%). Almost 80% of the students experienced the CA-PT as more difficult compared to the conventional PT. A total of 76% of students did not think they performed better on the CA-PT, and 24% of students thought that the CA-PT was better adjusted to their level (Figure 3). For approximately 90% of the students, the provided information on CAT was clear and they knew what to expect from the CA-PT. In response to the open question regarding their experience with the CA-PT (*n* = 422) the majority of comments were about: 1) missing the option to go back to the previous question (*n* = 112), 2) missing the question mark option (*n* = 87) and 3) it being more difficult to predict their performance level, leading to higher levels of insecurity and nervosity, and/or decreased motivation (*n* = 87).

## Discussion

To our knowledge, this study is the first to investigate test performance on a CA-PT compared to a conventional PT in an authentic setting with a large cohort of medical students at different study stages. A cross-over design was used in which all participating students were offered both a conventional PT and a CA-PT at a single timepoint and in an authentic examination setting. We found a strong correlation between test performance on the conventional PT and CA-PT. The CA-PT was administered without system performance issues, most students finished the CA-PT within two hours and students were motivated to perform well, despite the feeling that the CA-PT was more difficult.

The overall strong correlation between test performance on the conventional PT and CA-PT demonstrates that the CA-PT is able to reliably determine a students’ aptitude after a significantly shorter test. However, if we look at the correlation within the different year groups, the correlation was weaker in first-year students (r = 0.506). This may be explained by the fact that they had to answer all questions in the CA-PT and could not decide to use the question mark option in case they did not know the answer. More frequent use of the question mark in first year students lowers the total amount of answered questions and thereby the reliability of the conventional PT. In contrast, the larger amount of answered questions in the CA-PT ensures a more accurate, reliable score calculation, with a possibly larger variance in scores, which might explain the weaker correlation with the conventional PT in these students. Test reliability of the CA-PT is shown to be high for students across the full spectrum of ability, and thereby improves test reliability and quality especially for students in the first years of their study [11].

Overall, students were motivated, and engaged to perform well in the CA-PT. Although the students perceived the CA-PT as more difficult compared to the conventional PT, this was not reflected by poorer test performance. With respect to their attitude towards the CA-PT, our questionnaire data suggest that most students were persistent, had a mastery orientation, and adequate task management in the CA-PT. Additionally, the majority of students did not experience negative test-related motivation, and engagement, such as anxiety, self-handicapping (“*During this test I wasted time and was easily distracted*”), and disengagement (“*I often felt like giving up in this test*”). Our findings align with improved motivation for learning, and engagement with the test in the OAIPT project [23], and in elementary and secondary school students [22]. In contrast to this study [22], we did not find the specific factors self-efficacy and anxiety to be increased, although the open question reveals higher levels of insecurity and nervosity regarding performance level than answers to the closed questions suggest. Lower self-efficacy and increased insecurity may both be related to the degree of perceived control and the feeling that the items are well-matched to their performance level, as these factors are suggested to promote self-efficacy and diminish anxiety in CAT [34,35,36]. Nevertheless, these negative feelings were not accompanied by reduced motivation and engagement, which might be related to the fact that students felt challenged, well informed, knew what to expect, and were provided two opportunities to perform on the PT [22,37].

### Strengths and limitations

This multi-center study is the first to assess both test performance and test experience of a CA-PT in an authentic setting with medical students at different stages throughout the entire medical curriculum. The cross-over design and the short interval between the tests enabled us to compare performance within students at a given point in time, while the possible benefits for the students (best outcome counts for study credits) stimulated optimal test effort in both tests. However, the difference in delivery between the test formats, paper-based versus digital, might have influenced student performance depending on their preferences, though our experiences during the COVID-19 pandemic suggested that the effect on performance using different delivery formats is minimal (unpublished data). The difference in feeling of success, or certainty about their performance between the test formats might have had a psychological impact that differs between students. In the conventional PT, students usually experience a sense of how well they performed, derived from the proportion of items that they answered with certainty. In the CA-PT this sense is absent, as the number of wrong answers is approximately 50% for each individual. Our study sample was representative for all students participating in the conventional PT within the three medical schools where the study setting was facilitated, despite a slight overrepresentation of better performing students in two of the schools. Although the PT*first* and CA-PT*first* group were comparable in their performance on the CA-PT, students in the PT*first*-group performed slightly better on the conventional PT. Because students experienced decreased accuracy in estimating their performance on the CA-PT, or because they could review the questions of the conventional PT with the answer key directly afterwards, students in the PT*first*-group might have experienced less pressure to perform at their best in the CA-PT. Regardless, the effect of this group difference on the correlation was negligible (0.834 to 0.832). The study setting could only be facilitated in three of the seven medical centers. Still, the number of participants was large enough to leave our analysis of test performance uncompromised. Finally, two-thirds of the students who participated in the CA-PT did not return the questionnaire, which might have caused a bias regarding students’ opinion.

### Implications and future research

Taken together, our results support a broader application of the CA-PT in medical progress testing. As motivation, engagement and subjective test experience may affect students’ willingness to put effort in the test, and thereby influence their performance, it is relevant to shed light on these aspects [22,38]. Our finding that most students experienced the CA-PT as more difficult, and felt insecure about their performance, is important to take into consideration when preparing students for this new test format. Also, the responses to the open questions indicate that students find it difficult to switch to a new testing format, emphasizing the need for clear information, and practice opportunities. An interesting direction of future research could be the exploration of test performance, and student experiences over a longer period, as students continue getting accustomed to this testing format.

## Conclusion

In conclusion, this study shows that a CA-PT provides a reliable estimation of the students’ aptitude with a reduced test length in medical students. Students were motivated and engaged to perform well on the CA-PT, despite experiencing it as a more difficult test. Therefore, the implementation of a CA-PT in a wider context seems justified.

## Data Accessibility Statement

The data is available upon request, because we did not explicitly asked the students permission to share the data on an open repository in the informed consent.

## Additional Files

The additional files for this article can be found as follows:

10.5334/pme.1345.s1 Supplementary Material 1. Supplemental Table 1. Blueprint used in the conventional progress test (A) and in the (B) computer adaptive progress test.

10.5334/pme.1345.s2 Supplementary Material 2. Questionnaire Conventional versus Computer Adaptive Progress Test.
