# Supplementary material for: Computer Adaptive vs. Non-adaptive Medical Progress Testing: Feasibility, Test Performance, and Student Experiences
Source: Perspect Med Educ. 2024 Jul 26;13(1):406–16. doi: 10.5334/pme.1345 (PMC11276406; doi:10.5334/pme.1345)
Supplement: Supplementary Material 1. — Supplemental Table 1. Blueprint used in the conventional progress test (A) and in the (B) computer adaptive progress test. [file pme-13-1-1345-s1.pdf]

Supplemental Material

Supplemental Table 1. Blueprint used in the conventional progress test (A) and in the (B) computer adaptive progress test.

A.

Discipline	Number of questions
Anatomy	13
Biochemistry/Genetics/Histology/Molecular Cell Biology	18
Surgery	17
Dermatology/Ear, Nose, Throat/Ophthalmology	14
Epidemiology/Statistics	8
Pharmacology	9
Physiology	11
Geriatrics	8
Gynecology/Obstetrics	7
General Practice	20
Internal Medicine	26
Pediatrics	12
Metamedics	5
Neurology	7
Pathology/Immunology/Microbiology	10
Psychiatry/Psychology	12
Social Medicine	3
Total	200

B.

Discipline	Number of questions
Anatomy	7-8
Biochemistry/Genetics/Histology/Molecular Cell Biology	10-11
Surgery	10-11
Dermatology/Ear, Nose, Throat/Ophthalmology	8-9
Epidemiology/Statistics	4-5
Pharmacology	5-6
Physiology	6-7
Geriatrics	4-5
Gynecology/Obstetrics	4-5
General Practice	12
Internal Medicine	15-16

Pediatrics	7-8
Metamedics	3
Neurology	4-5
Pathology/Immunology/Microbiology	6
Psychiatry/Psychology	7-8
Social Medicine	1-2
Total	120
