# Supplementary material for: Computer Adaptive vs. Non-adaptive Medical Progress Testing: Feasibility, Test Performance, and Student Experiences
Source: Perspect Med Educ. 2024 Jul 26;13(1):406–16. doi: 10.5334/pme.1345 (PMC11276406; doi:10.5334/pme.1345)
Supplement: Supplementary Material 2. — Questionnaire Conventional versus Computer Adaptive Progress Test. [file pme-13-1-1345-s2.pdf]

Supplemental Material 2.
Questionnaire Conventional versus Computer Adaptive Progress Test

May 2022

Question 1*:* At which university do you study?

- o Amsterdam – University of Amsterdam
- o Amsterdam – Free University
- o Leiden University Medical Center
- o University Medical Center Groningen
- o Maastricht University Medical Center
- o Radboud University Medical Center Nijmegen
- o Erasmus Medical Center Rotterdam

Question 2: What is your student number?

Question 3: I performed in the conventional progress test (PT) on the 25th of May 2022.

- o Yes
- o No

The following questions concern the computer adaptive PT. The answers are given on a 7-point Likert Scale (1 = strongly disagree, 2 = disagree, 3 = somewhat disagree, 4 = either agree or disagree, 5 = somewhat agree, 6 = agree, 7 = strongly agree).

Motivation and Engagement

1. I did well in this test.
2. In this test I was very focused on understanding the questions and tasks.
3. This test was important.
4. I persisted in this test even when it was challenging or difficult.
5. In this test, I planned my answers and monitored my progress.
6. In this test I made good use of my time.
7. I was anxious in this test.
8. In this test I did not want to get a bad mark.
9. I do not think I had much control over how well I did in this test.
10. During this test I wasted time and was easily distracted.
11. I often felt like giving up in this test.

In the following questions we ask you to compare the computer adaptive PT to the earlier conventional progress tests. The answers are given on a 7-point Likert Scale (1 = strongly disagree, 2 = disagree, 3 = somewhat disagree, 4 = either agree or disagree, 5 = somewhat agree, 6 = agree, 7 = strongly agree).

<u>Subjective experience of the computer adaptive PT</u>

12. In comparison to the conventional PT, the computer adaptive PT was easy for me.
13. In comparison to the conventional PT, the computer adaptive PT was better adjusted to my level.
14. In comparison to the conventional PT, there were more questions in the computer adaptive PT where I expect to score points.
15. In comparison to the conventional PT, I have the feeling I performed better on the computer adaptive PT.

The following questions are open questions concerning the computer adaptive PT.

1. The provided information on computer adaptive testing was clear.

- Yes
- No, what was not clear? <*free text*>

2. I knew what to expect when I participated in the computer adaptive PT.

- Yes
- No; what surprised you? <*free text*>

3. This is what I encountered during the computer adaptive PT: <*free text*>
4. Any other remarks: <*free text*>
